# The predictive value of preoperative neutrophil-lymphocyte ratio (NLR) on the recurrence of the local pigmented villonodular synovitis of the knee joint

**DOI:** 10.1186/s12891-018-2258-5

**Published:** 2018-09-19

**Authors:** Guanglei Zhao, Jin Wang, Jun Xia, Yibing Wei, Siqun Wang, Gangyong Huang, Feiyan Chen, Jie Chen, Jingsheng Shi, Yuanqing Yang

**Affiliations:** Division of orthopaedic surgery, Huashan Hospital, Fudan University, Shanghai, 200040 China

**Keywords:** Pigmented villonodular synovitis, Recurrence, NLR

## Abstract

**Background:**

To explore and evaluate the predictive value of preoperative Neutrophil-lymphocyte ratio (NLR) on the recurrence of pigmented villonodular synovitis (PVNS) of the knee joint treated by arthroscopic surgery combining local radiotherapy.

**Methods:**

Sixty pathological-proven PVNS cases of the knee joint in our department from April 2006 to March 2017 were included. All of them are treated by arthroscopic synovectomy combined with adjuvant radiotherapy. The pre-operative hematological indexes such as c-reactive protein (CRP), erythrocyte sedimentation rate (ESR), NLR, Platelet-lymphocyte ratio (PLR) and Lymphocyte-monocyte ratio (LMR) were collected retrospectively and their relationship with postoperative recurrence was analyzed by using univariate and multivariate analysis, the receiver operating characteristic curves (ROC curve), the Kappa correspondence test and the Mc Nemar Chi-square test.

**Results:**

All 60 patients were followed up for a median of 52.8 months (7–138 months) and the recurrence rate is about 23.3% (14/60). There is a significant difference in NLR between the recurrent and non-recurrent group (*P* = 0.002). It had a certain correlation with postoperative recurrence (correlation coefficient *r* = 0.438, *P* = 0.001). The optimal thresholds in ROC curve were 2.42 (sensitivity 71.4%, specificity 78.3% respectively). which had predictive ability for recurrence after arthroscopic treatment.

**Conclusion:**

The preoperative NLR is an easy and cost-effective predictor for relapse in PVNS of the knee joint after the arthroscopic surgery combined with local radiotherapy, which is of profound significance to guide clinical work.

## Background

Pigmented villonodular synovitis (PVNS) is a rare and benign disease of the synovial membrane characterized by abnormal synovium proliferation and hemosiderin deposition. According to previously reported statistics, the incidence rate is about 1.8/100000. It mostly affects the knee joint [1, 2], but can occurs in any synovial joint including the hip, ankle and elbow joint [3, 4]. The PVNS can be classified into the localized and diffuse forms (LPVNS and DPVNS) [5]. The etiology of the PVNS is still unknown yet while it was regarded as an inflammatory disorder in the past decades. Some risk factors have been recognized such as the trauma, chronic inflammation, and abnormal lipid metabolism [6]. Furthermore, PVNS would invade the adjacent bone and soft tissue leading to the bone errosion and the deformity of the involved joint [7, 8]. The high recurrence rate and metastasis risk of PVNS had also been reported [9], hence, up to now, PVNS has been considered to be a neoplastic-like disorder of the synovium, with synovitis as a secondary reaction in PVNS [10].

Many researchers has found that some hematological parameters like CRP [11], platelet volume [12], NLR [13] and PLR [14] are closely related with the outcomes of many diseases such as inflammatory disease, autoimmune disease and neoplastic disease [15]. Notably, NLR, PLR or LMR are new, simple and cost-effective predictors for prognosis. A meta-analysis conducted by Zhang J [16] found that the elevated NLR has a close relationship with the poorer overall survival of colorectal cancer (HR = 1.92 95%CI = 1.57–2.34; *P* < 0.00001). Kaida T et al. [17] also reported that PLR is an independent predictive factor of recurrence beyond the Milan criteria after liver resection for patients with hepatocellular carcinoma (odds ratio, 2.55; 95% confidence interval, 1.17–5.49; *P* = 0.018). However, there have been no reports regarding the relationship between NLR, PLR or LMR and the relapse of PVNS of knee joint, in addition, no quantitative parameters now can predict the recurrence of the PVNS of knee joint effectively.

In this study, we retrospectively reviewed the clinical characteristics, blood indexes, and recurrence of sixty cases diagnosed with LPVNS of knee joint in our department. The purpose of the present study is to explore the relationship between the NLR, PLR or LMR and the recurrence of the PVNS of knee joint. We hypothesized that some of these parameters could be used as new predictors for the recurrence of LPVNS of knee joint which were treated by arthroscopic surgery and adjuvant radiotherapy.

## Methods

### Patients

Huashan Hospital follow-up system (HSFS) is a database established on the inpatient and outpatient database. The HSFS comprises medical complete records of inpatient and outpatient. Sixty patients histopathological diagnosed with knee PVNS (29 men and 31 women) are included in this study at orthopedic department of Huashan Hospital from April 2006 to March 2017. The study was approved by the Ethical Committee in Huashan Hospital. Baseline data including age, sex, height, weight, body mass index, X rays, Magnetic Resonance images (MRI), and results of laboratory tests including neutrophil, lymphocyte, monocyte, platelet counts, erythrocyte sedimentation rate (ESR), C-reactive protein(CRP) were collected from HSFS in the study. Chronic synovitis, such as rheumatoid synovitis and synovial chondromatosis were excluded. All enrolled participants had no history of trauma and surgery when initially visited our department. Repeated swelling, pain and limited joint function were the main clinical manifestation and the time between symptom onset and hospital admission ranged from 2 months to 8 years.

### Peroperative examinations

All patients received routine blood test and imaging examination including X rays and magnetic resonance image scan (MRI) before the surgery. In addition, the function of the involved knee joint before the surgery was assessed using Knee Society Score (KSS) and the Lyshoml Knee Score system.

### Surgical procedures

**Fig. 1:**
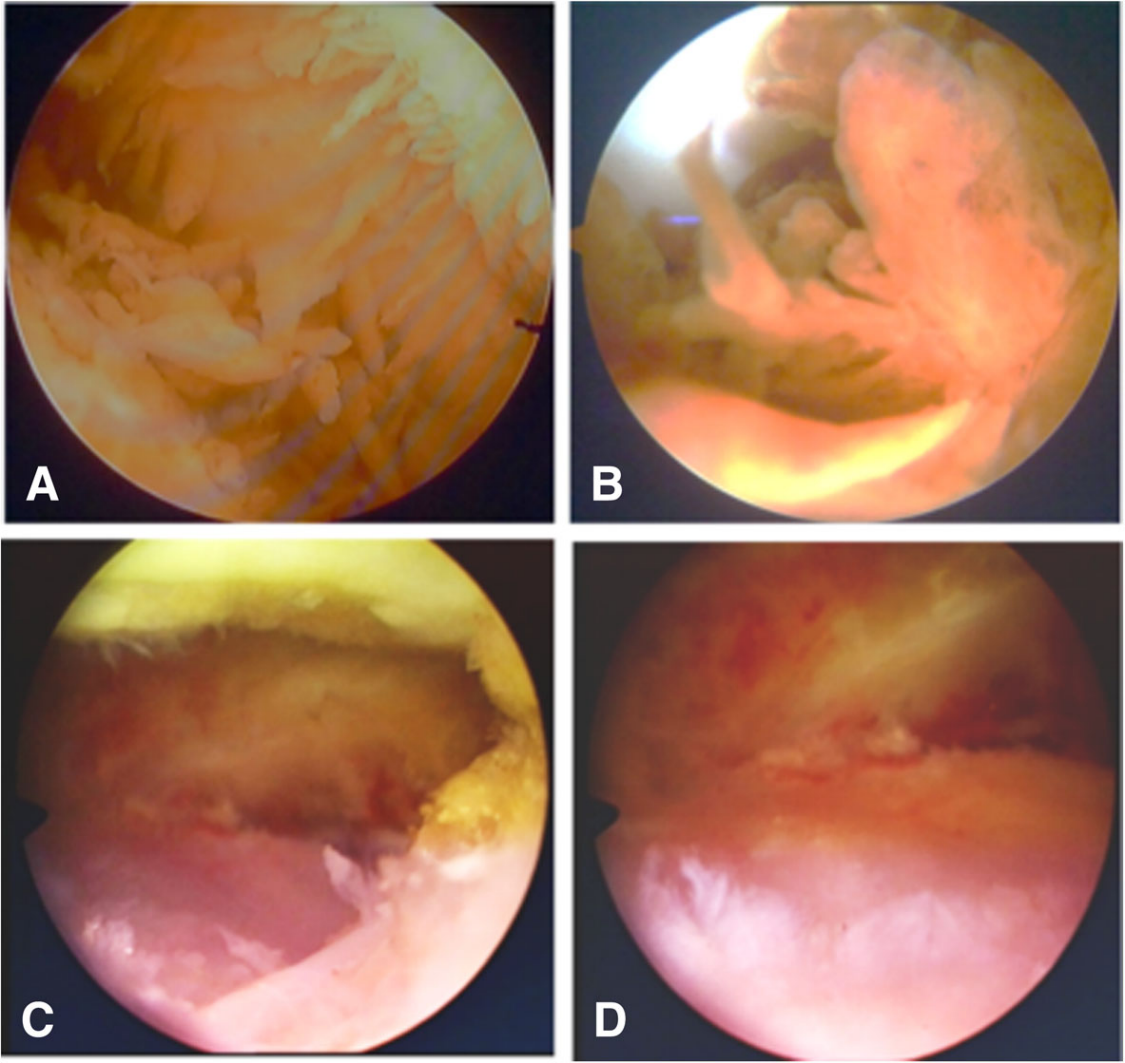
Macroscopic examination of the synovium before and after the arthroscopic synovectomy. (**a**-**b**) hypertrophied synovium with villous transformation and haemosiderin deposition before the surgery, (**c**-**d**) images of the knee joint after the synovectomy

All patients underwent comprehensive arthroscopic synovectomy in supine position under general anesthesia by an experienced surgeon. The pneumatic tourniquet (55-65 KPa) was used to stop the bleeding. Briefly, the standard anterolateral and anteromedial approaches were adopted and two 1 cm incisions was made on both sides of the patellar ligament in the front of the involved knee joint. Under the arthroscopic camera, the whole knee joint cavity was examined systematically according to the following order, suprapatellar bursa, patellofemoral joint, medial and lateral recess of the knee joint, tibiofemoral joint, meniscus and the anterior cruciate ligament. The abnormal synovial was removed as much as possible by using radiofrequency vaporization and shaving instruments. For PVNS lesions in the posterior joint, which was difficult to access from the anterior portals, the patients were placed prone. Posterolateral and posteromedial portals were used, and the PVNS lesions were removed as described before. Pathological examination of the abnormal synovium was performed routinely for each case. At the end of the procedure, surgeon would examine entire knee carefully again (Fig. 1). Finally, the wound was closed in layers.

### Postoperative management

On the first day after surgery, Wound compression and ice compress were used as usual and patients were encouraged to start range of motion (ROM) exercise after removing the thick dressing on the next day. Furthermore, the patients were permitted to conduct full weight-bearing as long as they can tolerate the pain and the non-steroid anti-inflammatory drugs or analgesics and decongestants are provided to relieve the pain. All patients discharged with a significant improvement in range of motion of the affected knee joint (0–90 degrees). Importantly, in order to reduce the local recurrence rate, patients were all advised to the Cancer Hospital of Fudan University for adjuvant radiotherapy 4–6 weeks after the surgery (average total dose is 2000 cGy to 3000 cGy, 10–15 times).

### Follow up

All 60 patients were followed through outpatient visit or telephone. The postoperative recurrence is defined as reoccurred joint swelling and pain 6 months after the treatment with typical appearance of PVNS on MRI images [18]. All patients were divided into the recurrent and non-recurrent group according to the outcome of the follow-up. The symptoms, Lysholm score and American KSS score were recorded before surgery and at final follow up evaluation or at the time of recurrence.

### Data analysis

The normal distribution of the data was assessed using the Kolmogorov–Smirnov test, the normally distributed data are presented as the means ± standard deviation (X ± SD) and M (P25, P75) for non-normally distributed variables. Clinical characteristics were compared between the recurrent group and non-recurrent group using Pearson’s X^2^ test for categorical variables and independent t-test for continuous variables. Spearman correlation was applied to assess correlations between relpase and the preoperative NLR. Receiver operating characteristic (ROC) analysis was used for evaluation of predictive markers in the recurrence of the knee PVNS. The optimal cutoff values of several markers including NLR, PLR, LMR that the best distinguished recurrent group from the non-recurrent group was determined with the maximum value of Youden’s index, which was calculated by sensitivity + 1-specificity [19]. The overall diagnostic accuracy and predictive ability were estimated based on the area under the curve (AUC) which is reported with its standard error. A multivariable analysis was performed with significant markers from ROC curves to determine which of them are independently associated with the relapse of knee PVNS. McNemar test and Kappa consistency test were also conducted to evaluate the effectiveness of the predictors. All Statistical analysis were performed with the SPSS software for windows (version 20.0; SPSS, Chicago, IL). *P* < 0.05 was considered statistically significant.

## Results

Sixty patients pathological diagnosed with PVNS were included (26 right knee, 34 left knee). The median age was 32 (range 14–75) years. On the MRI, the low signal intensity was presented on both T1 and T2 weighted images before the operation [18, 20]. The median duration of follow up was 52.8 (7–138) months and no complication of skin or wound infection was observed. Fourteen patients recurred and the median relapse time was 33.75 (20–51) months. Among the recurrent group, 3 patients underwent total knee arthroplasty and others received a second arthroscopy surgery.

Table 1 shows the clinical characteristics of the recurrent and non-recurrent group. The recurrent group showed higher NLR and CRP than non-recurrent group (P_NLR_ = 0.002, P_CRP_ = 0.04). There were no significant difference in other characteristics including PLR and LMR between the two groups (P_PLR_ = 0.23, P_LMR_ = 0.68).

**Table 1:** Clinical characteristics of all patients (* means statistical difference *P* < 0.05)

	Total	Recurrent	Non-recurrent	*P* value
Patients (n)	60	14	46	–
Age (years)	32.00 (26.25–47.00)	35.50 (28.75–50.00)	32.00 (26.00–48.25)	0.45
Gender (F/M)	31/29	6/8	25/21	0.45
Body mass index (kg/m^2^)	22.81 ± 1.81	23.31 ± 1.12	22.65 ± 1.95	0.12
Knee(L/R)	34/26	5/9	29/17	0.07
WBC (10^^^9/L)	6.57 (5.54–8.21)	7.01 (5.57–8.38)	6.57 (5.35–8.28)	0.51
ESR (mm/h)	14.00 (11.25–19.00)	16.50 (10.00–27.00)	13.50 (11.75–17.25)	0.208
CRP (mg/L)	13.30 (6.34–21.25)	17.31 (10.45–27.55)	12.51 (6.17–15.86)	0.04*
Neutrophils (10^^^9/L)	3.59 (2.92–4.53)	4.07 (3.22–5.36)	3.26 (2.52–4.51)	0.134
Platelets (10^9/L)	242.00 (189.75–281.25)	260.50 (235.00–282.25)	233.50 (180.75–278.50)	0.21
Lymphocytes (10^9/L)	1.87 (1.50–2.19)	1.83 (1.30–2.23)	1.87 (1.57–2.11)	0.69
Monocytes (10^9/L)	0.38 (0.32–0.46)	0.35 (0.32–0.46)	0.38 (0.32–0.46)	0.49
PLR	125.29 (105.04–152.58)	127.96 (104.05–233.92)	124.07 (105.71–148.65)	0.23
NLR	1.89 (1.40–2.50)	2.63 (2.05–2.84)	1.77 (1.36–2.25)	0.002*
LMR	4.76 (4.02–5.87)	4.75 (3.78–5.80)	4.76 (4.03–5.94)	0.68

The univariate and multivariate analysis were also performed with significant markers from ROC curves to determine which of them are independently associated with the diagnosis for relapse. The results indicates that NLR was significantly associated for prediction of relapse (odds ratio = 7.999, *P* = 0.017) (Table 2).

**Table 2:** Multiple logistic regression analysis of factors associated with relapse of PVNS of the knee joint. OR: odds ratio, 95% CI: 95% confidence intervals

	Univariate analysis			Multivariate analysis		
	OR	95% Cl	*p*-value	OR	95% Cl	*p*-value
Gender	1.587	0.475–5.307	0.453	1.217	0.176–8.411	0.842
Age	1.017	0.975–1.060	0.442	0.982	0.918–1.051	0.596
Body mass index	1.249	0.866–1.802	0.233	1.065	0.652–1.741	0.800
ESR	1.063	0.983–1.148	0.126	1.084	0.968–1.214	0.162
CRP	1.074	1.004–1.149	0.038^*^	1.113	1.004–1.235	0.043^*^
Neutrophils	1.467	0.884–2.434	0.138	1.063	0.478–2.363	0.823
NLR	6.595	1.877–23.170	0.003^*^	7.999	1.451–44.103	0.017^*^

*P values represent statistically significant differences

**Fig. 2:**
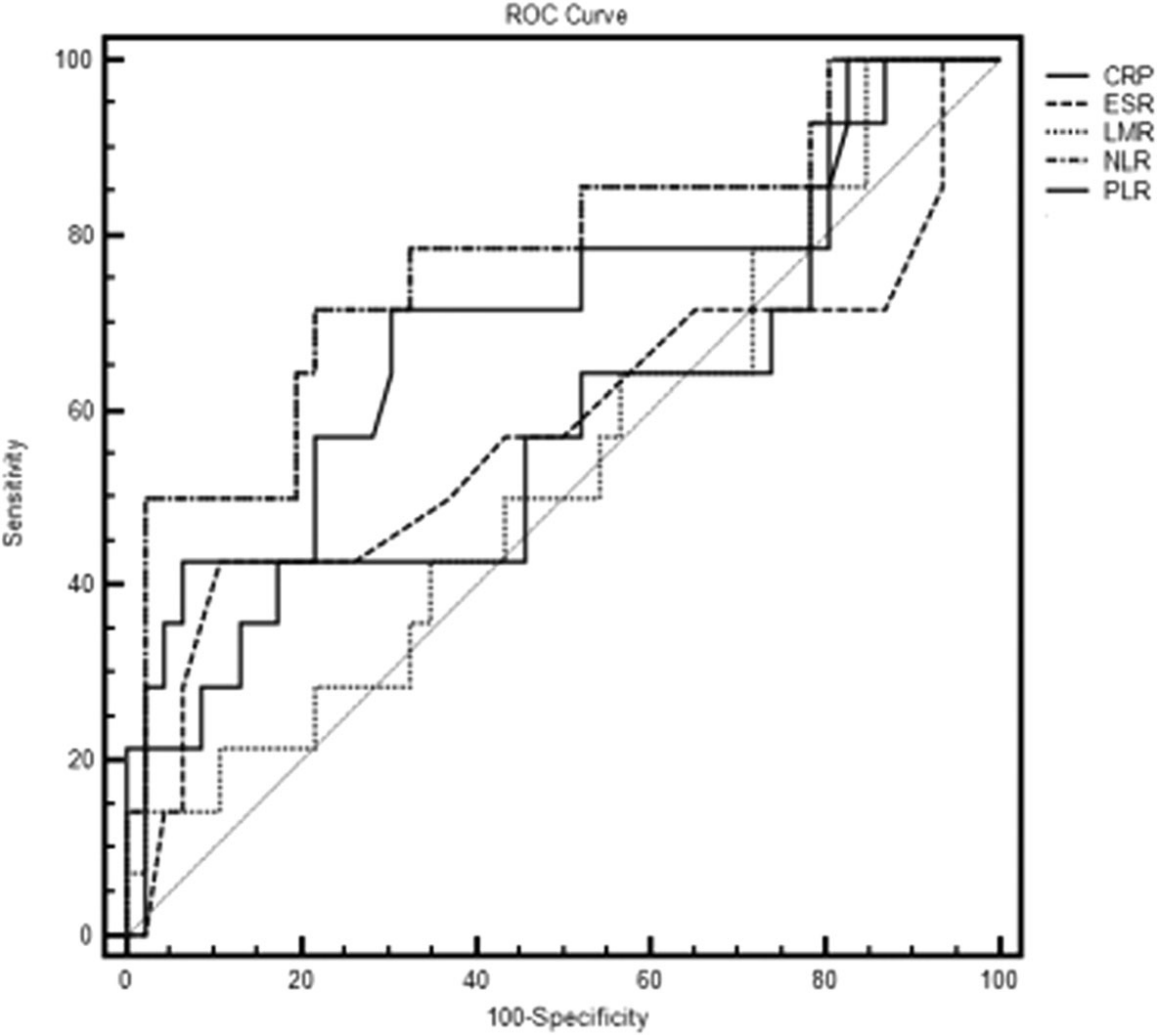
Receiver operating characteristic (ROC) curves for the neutrophil-to-lymphocyte ratio (NLR), Platelet-lymphocyte ratio (PLR), Lymphocyte-monocyte ratio (LMR) erythrocyte sedimentation rate (ESR), and C-reactive protein (CRP)

The optimal cutoff value that best distinguished recurrent from non-recurrent was determined at the maximum value, which was calculated by sensitivity + 1-specificity in the ROC curves. The ROC analysis of ESR, CRP, NLR, PLR and LMR showed the area under the curve (AUC) were 0.578 (95% CI = 0.443–0.704), 0.679 (95% CI = 0.545–0.793), 0.775 (95% CI = 0.649–0.873), 0.607 (95% CI = 0.473–0.731) and 0.537 (95% CI = 0.404–0.667) respectively (Fig. 2). Among the variables, the AUC of the NLR is the largest (AUC = 0.775, 95% CI = 0.649–0.873) and the optimal cut-off value is 2.42 for distinguishing the relapse which means when the NLR value is above 2.42 before treatment, the patient is more likely to have a relapse after the surgery. The Kappa test results (Kappa = 0.432, *P* = 0.001) and McNemar Chi-square test (*P* = 0.180) indicated that the NLR is valuable index for predicting the relapse.

The lyshoml and KSS score both improved in the two groups after the surgery though there is no significant difference before surgery and at the time of relapse in the recurrent group. Tables 3 and 4 shows that recurrent patients had poorer joint function than non-recurrent patients before the treatment did. In recurrent group, the knee functional score was higher at the time of relapse than that before operation while no significant difference was obtained. However, in non-recurrent group, most patients were satisfied with the greatly improved knee joint function and the difference in Lyshoml score and KSS score were statistically significant (*P* < 0.001) before surgery and at final follow up.

**Table 3:** The knee joint function in recurrent group

	Preoperative	Relapse / final follow up	*P* value
KSS clinical score	59.13 ± 7.59 (48–73)	64.88 ± 9.03 (50–77)	0.218
KSS function score	54.38 ± 18.20 (15–72)	60.38 ± 19.32 (20–78)	0.559
Lyshoml score	57.38 ± 12.91 (40–72)	62.38 ± 13.25 (43–76)	0.486

**Table 4:** The knee joint function in non-recurrent group (*means statistical difference *P* < 0.05)

	Preoperative	Final follow up	*P* value
KSS clinical score	68.12 ± 3.96	87.44 ± 5.20	< 0.001*
KSS function score	70.28 ± 4.88	88.92 ± 6.95	< 0.001*
Lyshoml score	66.96 ± 8.70	84.32 ± 9.72	< 0.001*

## Discussion

This is the first study to explore the relationship between preoperative hematological parameters and the recurrence of the PVNS of the knee joint. Our results indicate that the preoperative NLR is a useful predictive biomarker for the recurrence of PVNS. In our research, the NLR is significantly higher in the recurrence group (*P* = 0.002), besides, the NLR shows good correlation with the relapse and has a high sensitivity and specificity to predict the postoperative relapse with a cutoff value of 2.42.

The PVNS is a locally aggressive, neoplastic-like disorder of synovial tissue that it would cause functional deterioration of the involved joint. The recurrence rate of the PVNS is as high as 10–50% for the residual diseased synovium after the simple synovectomy [21]. Isart A et al. [22] also reported a high recurrence of PVNS (61.5%, 8/13) after arthroscopic synovectomy. Moreover, it is almost impossible to resect the pathological synovium completely by open access or arthroscopic therapy [23]. So the high recurrence rate is still an intractable clinical issue despite many reports have claimed that adjuvant local radiotherapy after the synovectomy may be a salvage option [24]. What’s more, the non-specific symptoms of PVNS at its early stage often contribute to a delay in establishing a diagnosis, and the joint arthroplasty is the curative surgery in the terminal stage cases with severe bone erosion. So the early diagnosis of relapse is necessary in PVNS after the first treatment for its high recurrence rate. However, there has been no effective quantitatively marker for the relapse. The pathological examination is the gold standard and the MRI is the mostly used radiological tool for monitoring the relapse of PVNS. However, the MRI is qualitative and expensive and not available in some remote districts, additionally, the pathological examination always needs a second invasive procedure. Hence, finding an easy, non-invasive and cost-effective biomarker for relapse is a great challenge for surgeons.

The NLR can be obtained simply from neutrophil and lymphocyte counts. In addition, the NLR is cheaper relatively and it is obtainable in most medical institution for the blood routine examination is one of the routine preoperative test. Sever studies have demonstrated that the NLR、LMR and PLR are significantly associated with the outcome of many diseases. For example, Kucuk A et al. [25] reported that the NLR is obviously higher in Ankylosing spondylitis (AS) patients compared to controls. (NLR = 2.47 ± 1.33, 1.72 ± 0.47, respectively, *P* < 0.0001). Additionally, there is a significant difference between the severe AS disease activity and the mild AS disease activity (NLR = 2.72 ± 1.41, 2.20 ± 1.19, *P* = 0.001). The results of the ROC analysis (cutoff value = 1.91, sensitivity 69%, specificity 54%) also proved that NLR is a simple and inexpensive marker to indicate disease activity in patients with AS in daily clinical practice. Lin JP et al. [26] found preoperative LMR is an independent prognostic factor for GC which can improve the predictability of individual survival and recurrence of gastric cancer. Moreover, the predictive value of the NLR, LMR or PLR have been found in more and more diseases including the diabetes, hypertension, pancreatic cancer and hepatocellular carcinoma [27–29].

To our knowledge, there have been no report about the NLR, LMR or PLR in predicting the recurrence of the knee PVNS. In our study, 60 patients diagnosed with LPVNS were enrolled. With respect to the gender ratio, our result (1.07, 31/29) was similar to those reported in Portugal (1.15) [30]. The BMI has no significant difference between two groups All participants received an arthroscopic synovectomy for the diagnosis of the LPVNS, and Xie et al. [15] found that no significant recurrence difference was identified between PVNS patients that were treated with open versus arthroscopic surgery (*p* = 0.78). In addition, the knee arthroscopic surgery, a minimally invasive surgery, is beneficial for fast recovery and shorter the hospitalization time. What’s more, the local adjuvant radiotherapy was applied to decrease the local relapse as much as possible. In a multicenter retrospective study, the recurrence rate in patients with knee PVNS is 24% (42/175) [2]. In our study, the recurrence rate is about 23.3% (14/60) which is consistent with the literatures. Among all hematologic indexes, Only CRP and NLR are significantly higher in recurrent patients (P_CRP_ = 0.04, P_NLR_ = 0.002). Other parameters like WBC, Neutrophils count and ESR are higher in recurrent group compared to controls, but don’t reach a statistical difference (P_WBC_ = 0.51, P_ESR_ = 0.208) which may implied that the PVNS is not a simple inflammatory disease. The results of ROC analysis and the multivariate analysis (Odds ratio = 7.99, 95% CI = 1.451–44.103, *P* = 0.017) indicated that the NLR may be a valuable marker for predicting relapse of knee PVNS after the treatment.

### Limitations

There were also some limitations in our study. Firstly, the number of the participants is relatively small for the low incidence of the PVNS, so the larger clinical researches with longer-term follow up are needed. VSecondly, all the patients enrolled in our study received the arthroscopic synovectomy and radiotherapy. Therefore, the effect of the surgical methods on the relapse is unknown. Thirdly, the mean duration of symptoms before the treatment is not included in our study for its inaccuracy.

## Conclusion

Our report suggests that the preoperative NLR is a valuable marker to predict the relapse of Knee PVNS treated by arthroscopic synovectomy combining radiotherapy. Our study indicated that these patients (preoperative NLR > 2.42) should be closely followed after the operation for the higher possibility of relapse. We hope this study could provide the orthopedic clinicians with a new method for predicting the postoperative recurrence of patients with PVNS.

## Abbreviations

AUC: Area under the curve
CRP: C-reactive protein
ESR: Erythrocyte sedimentation rate
HSFS: Huashan Hospital follow-up system
KSS: Knee Society Score
LMR: Lymphocyte-monocyte ratio
MRI: Magnetic Resonance images
NLR: Neutrophil-lymphocyte ratio
PLR: Platelet-lymphocyte ratio
PVNS: Pigmented villonodular synovitis
ROC: Receiver operating characteristic
ROM: Range of motion
